# RESPONSE TO THE AUTHORS “Intravaginal eletrical stimulation for bladder training method” by Cássio L. Z. Riccetto, 2021

**DOI:** 10.1590/S1677-5538.IBJU.2021.0817.1

**Published:** 2022-01-10

**Authors:** Cássio L. Z. Riccetto

**Affiliations:** 1 Universidade Estadual de Campinas Faculdade de Ciências Médicas Divisão de Urologia Feminina Campinas SP Brasil Divisão de Urologia Feminina, Faculdade de Ciências Médicas da Universidade Estadual de Campinas – UNICAMP, Campinas, SP, Brasil

*To the editor,*

While bladder training has long been a first-line treatment for women with overactive bladder syndrome (1, 2), its effectiveness as an isolated treatment is still somewhat controversial in the literature. In the prospective randomized study presented by Yildiz et al. (3), the combined use of intravaginal electrical stimulation plus bladder training determined a significant improvement in the overall success of the treatment of overactive bladder syndrome in women in comparison to Bladder training alone. We agree with the authors that the aim of their study was not to evaluate intravaginal electrical stimulation as an isolated method of treatment for an overactive bladder. In this regard, despite the widespread use of electrical stimulation techniques as pelvic floor rehabilitation strategies, its effectiveness as an isolated method is still controversial, as also referenced in the article (4). In fact, it is somewhat complicated to study the isolated effects of electrostimulation techniques for voiding dysfunctions, as the therapist's explanations about the treatment can also influence the patient's perception of their effects. We congratulate the authors for the initiative, highlighting the trend towards a multimodal approach to the treatment of overactive bladder syndrome in women (5).

The Author
